# Effects of Selected Diazotrophs on Maize Growth

**DOI:** 10.3389/fpls.2016.01429

**Published:** 2016-09-22

**Authors:** Medhin H. Kifle, Mark D. Laing

**Affiliations:** Discipline of Plant Pathology, School of Agricultural, Earth and Environmental Sciences, University of KwaZulu-NatalPietermaritzburg, South Africa

**Keywords:** diazotrophic bacteria, seedling growth, seed vigor chlorophyll level, maize, germination

## Abstract

Laboratory, greenhouse, and field experiments were conducted at the University of KwaZulu-Natal, Pietermaritzburg, South Africa in the 2010/2011 and 2011∖2012 seasons to study the effects of eight strains of diazotrophic bacteria on the growth and yield of maize. Maize seeds were treated with *Bacillus megaterium* (V16), *Pseudomonas* sp. (StB5, A3, A6, and A61), *Burkholderia ambifaria* (V9), *Enterobacter cloacae* (L1) and *Pantoea ananatis* (LB5), aiming to stimulate plant growth, and maintain or increase yields while reducing the need for N fertilization. All the diazotrophic bacteria increased germination of maize seed, and *Pseudomonas* sp. (StB5) and *B. megaterium* (V16) significantly increased shoot length. *Pseudomonas* sp. (StB5), *B. megaterium* (V16), *E. cloacae* (L1), *B. ambifaria* (V9), and *Pseudomonas* sp. (A3) very significantly increased root length and seed vigor index. Under greenhouse conditions, plants treated with diazotrophic bacteria developed more leaf chlorophyll and greater dry weight, albeit not significantly (n.s.). In a field trial in 2010/2011, application of the best five diazotrophic bacteria, with or without 33% N-fertilizer, had no significant effect on germination, grain yield, dry weight, plant height and leaf chlorophyll. In the 2011/2012 growing season, at 60 days after planting (DAP), all the diazotrophic bacteria increased plant dry weights to equal that of the fertilized control (33%N-fertilizer) (n.s.). After inoculation with the diazotrophs alone increased plant heights (n.s.), and chlorophyll contents (n.s.). With the addition of 33%N-fertilizer at planting, the diazotrophs still caused increases of chlorophyll content relative to the control with 33%N (n.s.). It may be concluded that the tested diazotrophs alone may be beneficial for use on maize growth.

## Introduction

Maize is the most important staple food in South Africa (Du-Plessis, 2003). Correct application of inputs are necessary for successful maize production. However, poor soil fertility, drought, and disease are among the constraints to production (Lynch, 2007). Nitrogen fertilization is an essential input required for high yields. However, a substantial proportion of the N fertilizers applied to crops are lost through gaseous emissions, denitrification and leaching of nitrates into ground water (Sekhon, 1995), which impacts negatively on the environment (Hagin and Lowengart, 1995; Rejesus and Hornbaker, 1999).

Bacteria in the rhizosphere of plants that exert beneficial effects are called plant growth promoting rhizobacteria (PGPR; Kloepper et al., 1989). PGPR promote growth directly by providing nutrients or enhancing nutrient uptake, and indirectly by suppressing plant pathogens (Vessey, 2003; Ahmad et al., 2008). Use of microbial inoculants to enhance growth and increase yields of crops has attracted the interest of many researchers (Bashan, 1998; Dobbelaere et al., 2003; Kennedy et al., 2004; Cakmakçi et al., 2006; Nain et al., 2010; Nadeem et al., 2014). Many PGPR are also N-fixing bacteria (diazotrophs), and these include the genera *Azospirillum, Azotobacter, Bacillus, Pseudomonas*, and *Serratia* (Bashan et al., 2004). Their ability to fix nitrogen probably makes the organisms better adapted to live in the rhizosphere. A widely studied diazotrophic bacterium, *Azospirillum brasilense* promotes plant growth not only through N-fixation but also through other mechanisms such as phyto-hormones (Spaepen et al., 2009). Increases in the growth and yield of important crops in response to inoculation with diazotrophic bacteria have been reported by Okon and Labandera-Gonzalez (1994), Bashan et al. (2004), and Kennedy et al. (2004). Strains of *Bacillus* spp., *Pseudomonas* spp., *Enterobacter* spp., *Burkholderia* spp., and *Pantoea* spp. may affect seed germination, seedling growth and yield (de Freitas, 2000; Vázquez et al., 2000; Liu et al., 2006). Inoculation of plants with strains of *Enterobacter* spp. may result in significant increases in various growth parameters, such as increases in plant biomass, nutrient uptake, N content, plant height, leaf size and root length of cereals (Bashan et al., 2004), and some can also be used as biocontrol agents (Duponnois et al., 1999).

The objective of this study was to evaluate whether selected diazotrophic bacteria could enhance growth parameters of maize under greenhouse and field conditions when applied by themselves or with a limited initial topdressing with N-fertilizer.

## Materials and Methods

### Seed Source

Seeds of white maize of the cultivar, Mac’s Medium Pearl (an open pollinated variety) were purchased from McDonalds Seeds (McDonalds Seed Company (Pty) Ltd., Pietermaritzburg, South Africa) surface sterilized with 1% sodium hypochlorite for 5 min and washed five times with sterilized distilled water.

### Seed Treatment

Eight diazotrophic bacterial strains were previously selected for their N fixing ability: *Bacillus megaterium* (V16), *Pseudomonas* sp. (StB5, A3, A6, and A61), *Burkholderia ambifaria* (V9), *Enterobacter cloacae* (L1), and *Pantoea ananatis* (LB5) (Kifle and Laing, 2016). These bacteria were cultured in tryptic soy broth (TSB) (Merck) for 48 h at 28 ± 2°C in a shaker at 150 rpm.

Final pellets of bacterial cells were diluted with sterile distilled water and cell numbers were adjusted to 10^8^cfu mL^-1^, as determined by serial dilution and plating. Two grams of a sticker, gum arabic, were dissolved in 100 mL of bacterial suspension, stirred, and allowed to stand for 1 h to create a homogeneous suspension. Seed coating took place in a plastic bag. Maize seed (200 g) was placed in a plastic bag and 20 ml of the bacterial sticker suspension was added (0.1 mL g^-1^ of seeds). The bag was shaken for 2 min, until all the seeds were uniformly coated. Cell count was approximately 10^6^ CFU per seed. The bag was opened and the seed spread onto paper towels and air dried overnight.

### Germination Bioassay

Germination tests were performed using a paper towel method following the procedures described by Gholami et al. (2009). Seed treated as above was compared with an Un-treated Control (treated with sterile distilled-water amended with gum arabic). Each treatment was replicated three times. Seeds were germinated in a growth chamber at 28°C. After 5 days, the number of germinated seeds was counted, and root and shoot length of individual seedling was measured. Vigor index were determined with the following formula: seed vigor index = [(mean root length + mean shoot length) X germination %] (Abdul-Baki and Anderson, 1973).

### Greenhouse Experiment

A greenhouse experiment was carried out at University of KwaZulu-Natal, Pietermaritzburg, South Africa in an environmentally controlled greenhouse with temperatures varying between 26 and 28°C. The experiment was arranged in a randomized complete blocks design with 10 treatments and three replicates, and nine plants per replicate. The treatments were: (i) seeds treated with eight diazotrophs, (ii) Un-treated Control with no N-fertilizer, and (iii) Un-treated Control with 33%N-fertilizer. Plants were grown in 200 mm diameter pots containing 2 kg of growing medium (composted pine bark). Each pot was planted with five seeds, and thinned to three plants per pot. Leaf chlorophyll content was measured using a hand held chlorophyll content meter (CCM-200 plus, Optic-Science, 8 Winn Avenue, Hudson, NH 03051, USA). Chlorophyll readings were taken on the midpoint of the youngest fully expanded leaf and on the ear leaf of five leaves selected at random at 8 weeks after planting. Eight weeks later. Dry biomass was obtained by harvesting whole maize plants at 8 weeks, and oven-drying them for 72 h at 70°C.

### Field Experiments

Field experiments were conducted at Ukulinga, a research farm of the University of KwaZulu-Natal, Pietermaritzburg, South Africa (29° 24′ E; 30° 24′ S). Soils at the site are 55% clay content. Each plot was 8.7 m long × 3.75 m wide, and consisted of six rows with a 0.75 m inter row spacing. Five diazotrophs were selected from the greenhouse trials: *B. megaterium* (V16), *Pseudomonas* spp. (StB5), *B. ambifaria* (V9), *E. cloacae* (L1), and *P. ananatis* (LB5). Seeds were treated with the diazotrophs before planting, as above, and hand-planted on the 24th of November 2010, the 10th of November 2011. Plots were irrigated as needed. Weeds were controlled by hand weeding. Germinated seedlings were counted 14 days after planting (DAP) and the germination percentage calculated. Chlorophyll readings were taken on the midpoint of the youngest fully expanded leaf and on the ear leaf at 10 weeks after planting. Ten leaves were measured at random in each plot. Plant height was measured by randomly selecting 10 plants in each plot, and measuring the distance from the ground to the stem tip. Cobs were harvested at maturity to secure yield data. Dry biomass was obtained by harvesting three whole maize plants from each plot at 30, 60, and 90 DAP, and oven-drying them for 72 h at 70°C.

### Fertilizer Application

In the greenhouses study fertilization was as follows: maize seedlings treated with diazotrophic bacterial strains were watered every day with 500 mL nutrient solution containing: 0.11 mL L^-1^ H_3_PO_4_; 0.13 g L^-1^ KOH; 0.14 g L^-1^ K_2_SO_4_; 0.74 g L^-1^ CaCl_2_.2H_2_O; 0.10 g L^-1^ MgSO_4_.7H_2_O and 0.02 g L^-1^ of micronutrients (MICROPLEX^®^) [Ocean Agriculture (Pty) Ltd, P.O. Box 741, Mulders drift 1747, South Africa]. There were two controls: one was untreated and supplemented with a soluble fertilizer NPK (3:1:3 [38] complete) which was purchased from Ocean Agriculture (Pty) Ltd, P.O. Box 741, Mulders drift 1747, South Africa and applied at a rate of 0.33 g L^-1^. The second was untreated control and supplemented with MICROPLEX^®^ nutrient solution. In the field, lime ammonium nitrate (LAN) purchased from (Sasol Nitro, division of Sasol chemical industries Limited, P.O. Box 5486, Johannesburg 2000, South Africa) applied at a rate of 165 kg ha^-1^, Superphosphate purchased from Omnia Fertilizer Group (Pty) Ltd, P.O. Box 69888, Bryanston 2021, South Africa) was applied at a rate of 100 kg ha^-1^. According to the soil analysis result the field has enough potassium (K) therefore no K was recommended.

### Experimental Design and Analysis

One way ANOVA was used to analyze data from greenhouses and factorial analysis for the field using GenStat^®^ 14th edition. *F*-values for main treatment effects and their interaction were considered significant at 5%. When a particular factor or an interaction of factors significantly influenced a variable, means were separated using Duncan’s multiple range tests at 5% probability level.

## Results

### Germination Test

In order to analyze the effects of diazotrophic bacteria as bio-inoculants for maize growth, selected diazotrophic bacteria, *B. megaterium* (V16), *Pseudomonas* spp. (StB5, A3, A6, and A61), *B. ambifaria* (V9), *E. cloacae* (L1) and *P. ananatis* (LB5), were tested and caused significant increases in % germination compared to the untreated control (**Table 1**). *Pseudomonas* spp. (StB5) and *B. megaterium* (V16) significantly increased shoot length. Moreover, seeds treated with diazotrophic bacteria, *Pseudomonas* spp. (StB5, A3), *B. megaterium* (V16), *E. cloacae* (L1), and *B. ambifaria* (V9) significantly (*P* < 0.001) increased root length and enhanced the seed vigor index of maize seedlings (**Table 1**).

**Table 1:** Effect of N-fixing bacterial isolates on the growth of maize seedlings.

Treatment	Germination %	Shoot length (cm)	Root length (cm)	Seed Vigor Index
Control	34.87 a	7.83 a	2.67 a	366.14
*Pseudomonas* sp. (A3)	59.56 b	10.17 ab	13.00 bc	1380.01
*Pseudomonas* sp. (A61)	65.15 b	9.33 ab	6.67 ab	1042.40
*Pantoea ananatis* (LB5)	71.18 b	8.33 a	7.33 ab	1114.68
*Pseudomonas* sp. (StB5)	71.85 b	21.83 c	16.00 c	2718.09
*Pseudomonas* sp. (A6)	77.74 b	9.00 ab	7.17 ab	1257.06
*Bacillus megaterium* (V16)	79.24 b	17.50 b	19.17 c	2905.73
*Enterobacter cloacae* (L1)	79.54 b	15.33 ab	15.67 c	2465.74
*Bacillus ambifaria* (V9)	82.70 b	15.67 ab	17.33 c	2729.10
CV%	19.80	33.30	30.70	
Lsd	24.11	7.845	6.485	
Sed	11.48	3.734	3.087	
*P*	0.01	0.003	<0.001	

*Means with the same letter in the same column are not significantly different at P ≤ 0.05.*

### Effect of Selected Diazotrophs on Growth of Maize

All the diazotrophic bacteria without N-fertilizer caused significant increases in dry weight compared to untreated control without N-fertilizer (**Table 2**). Although, it was not statistically significant, *B. megaterium* (V16), *Pseudomonas* sp. (A6 and A61) with 33%N-fertilizer caused increases in dry weight of 46.4, 23.9, or 6.7%, respectively, over the untreated control with 33%N-fertilizer (**Table 2**). Although, it was not statistically significant, *Pseudomonas* spp. (StB5) and *B. megaterium* (V16) with 33%N fertilizer caused 15.5 and 18.3% increases in chlorophyll content, respectively, over the untreated control with 33%N-fertilizer. Unexpectedly, plants treated with *Pseudomonas* sp. (A6) and 33%N fertilizer showed a statistically significant decrease in chlorophyll content (**Table 2**).

**Table 2:** Greenhouse study on the effect of diazotrophs on maize growth.

N-fertilizer (%)	Isolates	Chlorophyll (CCI)	Dry weight (g)
0	Control	3.58 [0.55] a	1.67 [0.22] a
	*Pseudomonas* sp. (StB5)	3.75 [0.57] a	7.38 [0.85] defg
	*Bacillus ambifaria* (V9)	4.29 [0.63] a	6.31 [0.81] cdef
	*Pantoea ananatis* (LB5)	4.32 [0.64] a	4.97 [0.69] bcd
	*Bacillus megaterium* (V16)	4.48 [0.65] a	5.75 [0.76] bcde
	*Pseudomonas* sp. (A3)	4.52 [0.65] a	4.40 [0.64] bc
	*Pseudomonas* sp. (A6)	4.59 [0.66] a	3.77 [0.57] b
	*Enterobacter cloacae* (L1)	4.68 [0.66] a	4.84 [0.68] bcd
	*Pseudomonas* sp. (A61)	4.84 [0.68] a	4.60 [0.66] bcd
33	Control	8.76 [0.94] c	10.93 [1.03] ghi
	*Pseudomonas* sp. (StB5)	10.12 [1.00] c	9.66 [0.98] fgh
	*B. ambifaria* (V9)	8.78 [0.94] c	8.68 [0.93] efgh
	*P. ananatis* (LB5)	8.62 [0.93] c	9.68 [0.97] fgh
	*B. megaterium* (V16)	10.36 [1.02] c	16.00 [1.19] i
	*Pseudomonas* sp. (A3)	7.88 [0.90] c	11.02 [1.02] ghi
	*Pseudomonas* sp. (A6)	6.50 [0.80] b	13.55 [1.12] hi
	*E. cloacae* (L1)	8.73 [0.93] c	9.93 [0.99] fgh
	*Pseudomonas* sp. (A61)	8.21 [0.91] c	11.66 [1.06] hi
		*P* = 0.02 [0.025]	*P* = 0.004 [0 < 001]
		CV% = 15.80 [8.7]	CV% = 28.5 [12]

*Means with the same letter in the same column are not significantly different at P < 0.05, values in parenthesis are transformed data using log base 10*.

### Growth Parameters of Maize under Field Conditions in the Year 2010–2011

Application of five of the diazotrophic bacteria as seed treatment resulted in increases in seed germination by 57.5–74.2% over the untreated control with or without added N (**Table 3**). Dry weights did not increase uniformly as a result of the application of diazotrophs, with or without 33%N application. The diazotrophs, with or without 33%N- fertilizer, caused no significant increases in grain yield, dry weight, plant height and chlorophyll content (**Tables 3** and **4**), although, *E. cloacae* (L1) and *P. ananatis* (LB5) with 33%N fertilizer caused 55.9 and 59.8% increases (n.s.) in dry weight, respectively, over the untreated control with 33%N at 60 DAP (**Table 3**). Unexpectedly, *P. ananatis* (LB5) induced a significant decrease in grain yield when applied with 33%N- fertilizer, despite having increased germination significantly by 25.8% (although yield was taken from the same number of plants per plot).

**Table 3:** Effect of diazotrophic bacteria on maize growth in the 2010/2011 season.

Treatments		% germination	Dry weight (g) 2010/2011 season			Yield (kg ha^-1^)
N-Fertilizer (%)	Bacteria		30 DAP	60 DAP	90 DAP	
	Control	35.3 a	18.85 a	143 bc	796.3 a	368.84 a
0	*Enterobacter cloacae* (L1)	58.9 abc	13.99 a	149 bc	588.8 a	432.18 ab
	*Bacillus megaterium* (V16)	55.5 abc	14.03 a	129 ab	686.6 a	460.79 abc
	*Pantoea ananatis* (LB5)	61.5 bc	15.73 a	124 a	783.4 a	448.53 abc
	*Bacillus ambifaria* (V9)	69.5 c	17.12 a	146 c	731.6 a	468.97 abc
	*Pseudomonas* sp. (StB5)	56.0 abc	17.55 a	145 bc	726.5 a	453.64 abc
33	Control	43.1 ab	17.01 a	160 c	730.4 a	509.83 bc
	*E. cloacae* (L1)	67.2 bc	18.26 a	159 c	769.0 a	457.73 abc
	*B. megaterium(*V16*)*	55.5 abc	19.49 a	155 bc	721.2 a	430.14 ab
	*P. ananatis* (LB5)	68.9 c	17.48 a	153 bc	681.8 a	382.12 a
	*B. ambifaria* (V9)	57.8 abc	15.91 a	161 c	666.6 a	430.14 ab
	*Pseudomonas* sp. (StB5)	53.7 abc	17.23 a	161 c	720.0 a	443.42 ab
		*P* = 0.056	*P* = 0.326	*P* = 0.764	*P* = 0.89	*P* = 0.055
		CV% = 15.8	CV% = 18	CV% = 9.4	CV% = 20.9	CV% = 13.3

*Means with the same letter in the same column are not significantly different at P ≤ 0.05.*

**Table 4:** Effect of bacterial isolates on plant height and chlorophyll content in the 2010–2011 season.

Treatments		Plant height (cm) 2010/2011			Chlorophyll content index	
N-fertilizer (%)	Bacteria	30 DAP	60 DAP	90 DAP	30 DAP	60 DAP
0	Control	44.91 a	70.59 a	176.5 a	39.95 a	69.63 a
	*Enterobacter cloacae* (L1)	46.37 a	68.18 a	173.3 a	46.37 a	80.16 a
	*Bacillus megaterium(*V16*)*	46.82 a	62.85 a	154.2 a	47.92 ab	75.55 a
	*Pantoea ananatis* (LB5)	48.94 a	69.19 a	159.5 a	48.94 ab	79.19 a
	*B. ambifaria* (V9)	49.81 a	66.06 a	171.3 a	45.90 a	76.49 a
	*Pseudomonas* sp. (StB5)	59.30 a	67.15 a	179.2 a	59.30 b	77.15 a
33	Control	56.36 a	68.89 a	176.8 a	60.42 b	79.91 a
	*E. cloacae* (L1)	46.26 a	69.89 a	165.3 a	48.98 ab	78.41 a
	*B. megaterium(*V16*)*	47.82 a	67.25 a	186.7 a	47.82 ab	77.25 a
	*P. ananatis* (LB5)	46.21 a	63.38 a	168.0 a	46.21 a	73.38 a
	*B. ambifaria* (V9)	44.69 a	68.27 a	178.2 a	44.69 a	81.03 a
	*Pseudomonas* sp. (StB5)	47.41 a	69.63 a	186.0 a	47.41 ab	78.61 a
		*P* = 0.629	*P* = 0.694	*P* = 0.116	*P* = 0.042	*P* = 0.179
		CV% = 17.1	CV% = 8.7	CV% = 7.4	CV% = 16.1	CV% = 6.7

*Means with the same letter in the same column are not significantly different at P ≤ 0.05.*

### Effects of Diazotrophs on Maize Growth in the (2011–2012) Season

Application of *B. megaterium* (V16), *Pseudomonas* sp. (StB5), *B. ambifaria* (V9), and *E. cloacae* (L1) as seed treatment without added N-fertilizer caused high significant increases in %germination compared to untreated control without N-fertilizer (**Table 5**). At 30 or 90 DAP, the selected diazotrophs without added N-fertilizer caused no significant increases in dry weight compared to untreated control without N (**Table 5**). At 60 DAP, all the diazotrophic bacteria performed significantly higher in dry weight compared to untreated control without N and stood equivalent to untreated control with 33%N-fertilizer (**Table 5**). No significant increases in yield were observed due to the treatment by these diazotrophic bacteria without added N-fertilizer. *Pseudomonas* sp. (StB5), *E. cloacae* (L1), *B. ambifaria* (V9), and *B. megaterium* (V16) without added N-fertilizer at 30, 60, or 90 DAP caused highly significant increases in plant height compared to untreated control without N-fertilizer (**Table 6**). However, these diazotrophs with 33%N, resulted no significant increases in height compared to untreated control with 33%N (**Table 6**). Although, it was not statistically significant, the selected diazotrophs without added N-fertilizer caused 44.6–77.7% increases in chlorophyll content over the untreated control without N-fertilizer, and 15.4–28.8% increases over untreated control with 33%N. (**Table 6**). However, with 33%N these diazotrophs caused only 15.4–28.8% increases in chlorophyll content over untreated control with 33%N.

**Table 5:** Maize growth parameters in the 2011/2012 season.

Treatments		% germination	Dry weight (g) in season 2011/2012			Yield (kg ha^-1^)
N-fertilizer (%)	Bacteria		30 DAP	60 DAP	90 DAP	
0	Control	37.3 a	14.3 a	56.5 a	363 a	301 a
	LB5	44.6 ab	14.8 a	98.1 b	498 abc	309 ab
	V9	59.0 cd	15.8 a	96.9 b	542 abc	307 ab
	V16	54.7 bc	15.9 a	109.3 bcd	465 ab	309 ab
	L1	64.7 cde	16.2 a	102.8 bc	513 abc	302 a
	StB5	74.6 ef	17.2 ab	97.3 b	531 abc	337 abc
33	Control	82.7 fg	26.3 bc	117 bcd	565 bc	415 bc
	LB5	63.0 cde	16.7 ab	113.7 bcd	492 abc	403 abc
	V9	85.3 fg	20.0 ab	126.7 d	542 abc	363 abc
	V16	54.7 bc	19.0 ab	122.4 cd	579 bc	440 c
	L1	74.0 def	16.9 ab	116.9 bcd	663 cd	405 abc
	StB5	88.0 fg	18.9 ab	122.0 cd	601 bcd	425 c
		*P* < 0.001	*P* < 0.001	*P* < 0.001	*P* < 0.001	*P* < 0.001
		CV% = 28.5[12]	CV% = 21.4	CV% = 6.3	CV% = 10.5	CV% = 0.92

*Means with the same letter in the same column are not significantly different at P ≤ 0.05.*

**Table 6:** Effect of bacterial isolates on plant height and chlorophyll content in the 2011/2012 season.

Treatments		Plant height (cm)			Chlorophyll content index	
N%-fertilizer	Bacteria	30 DAP	60 DAP	90 DAP	30 DAP	60 DAP
0	Control	11.03 a	47.67 a	102.7 a	21.22 a	18.17 a
	LB5	17.53 ab	51.20 ab	150.7 b	30.68 ab	42.93 abc
	StB5	24.20 bc	63.27 cd	157.3 bc	34.95 b	30.93 ab
	L1	24.47 bc	57.90 bc	158.7 bcd	31.28 ab	37.87 abc
	V16	27.00 c	66.67 cd	152.3 b	39.63 b	41.60 abc
	V9	28.60 c	64.07 cd	159.0 bcd	37.70 b	36.77 abc
33	Control	41.37 de	82.43 f	172 cde	39.65 b	45.28 abc
	LB5	36.57 d	71.5 de	162.7 bcd	35.45 b	54.13 bc
	StB5	39.83 de	83.83 f	173.7 cde	37.32 b	53.60 bc
	L1	36.27 d	78.8 ef	174 cde	38.05 b	60.74 bc
	V16	40.17 de	81.5 f	181.7 ef	40.63 b	56.90 bc
	V9	39.83 de	83.17 f	176.7 de	35.77 b	59.92 bc
		*P* < 0.001	*P* < 0.001	*P* < 0.001	*P* < 0.001	*P* = 0.54
		CV% = 14.1	CV% = 7.2	CV% = 5.9	CV% = 9.4	CV% = 20

*Means with the same letter in the same column are not significantly different at P ≤ 0.05.*

## Discussion

Maize (*Zea mays* L.) is among the most widely cultivated crops in South Africa (Du-Plessis, 2003). Nitrogen is the most required plant nutrient in crop production (Fageria and Baligar, 2005), but is often the limiting nutrient for maize produced in the tropics (Osmond and Riha, 1996). A single mature maize plant takes up approximately 8.7 g of N from the soil (Du-Plessis, 2003). Maize production has an impact on environmental issues, including climate change because of the use of fossil energy to produce synthetic N-fertilizer, and via the impact of leached N polluting water supplies.

According to the South Africa Grain Information Services (SAGIS), in the year 2015, maize production from non-commercial agriculture was 442,200 and 231,600t for white and yellow maize, respectively (Dredge, 2016). About 60% of the maize production in the non-commercial sector was planted in the Eastern Cape where poor soil fertility is a challenge (Materechera, 2010; Weber et al., 2012). These farmers apply no or little fertilizers to their crop (Kibirige, 2014). Therefore, resource-poor farmers may benefit from the use of diazotrophic bacteria as bio-inoculants because this technology may provide for the N needs of their maize crops.

In the current study, inoculation of diazotrophic bacteria as a maize seed treatment, with or without the addition of some N-fertilizer, increased germination, root and shoot length and enhanced the seed vigor index of treated maize seedlings. Among the tested diazotrophs, StB5 (*Pseudomonas* sp.) and V16 (*B. megaterium)* enhanced shoot length and seed vigor index. Strains StB5 and A3 (*Pseudomonas* sp.), V16 (*B. megaterium)*, L1 (*E. cloacae*), and V9 (*B. ambifaria*) enhanced root length and seed vigor index. This suggested that the tested diazotrophic bacteria have some plant growth promoting features that enhanced germination, root and shoot length and the seed vigor index of maize. A previous study has shown that inoculation of maize with *Pseudomonas* sp. Strain CDB 35 and *Serratia marcescens* Strain EB 67 enhanced biomass, root and shoot length and seed vigor index (Hameeda et al., 2008). In the greenhouse study here, the tested diazotrophic bacteria without N-fertilizer enhanced maize dry weight. However, when 33%N was added, only *B. megaterium* (V16) and *Pseudomonas* sp. (A6) increased dry biomass. In a previous study, improved plant biomass was observed when maize seeds were inoculated with a strain of *Pseudomonas* sp. (Sandhya et al., 2010). In another study, increases in plant height, plant biomass, and root length was observed when maize was inoculated with *Burkholderia* sp. Strain CC-Al74 (Young et al., 2013).

In the field, in the 2010/2011 season, seed treatments with selected diazotrophs resulted in increases in seed germination. However, they caused no significant increases in grain yield, dry weight, plant height and chlorophyll content when compared to the untreated control. This may have been due to high competition from the indigenous soil microflora, given that success of microbial inoculation depends on the colonization and competitive ability of the inoculants (Afzal et al., 2012). Plant roots exudates, colonization of roots by other bacteria, and soil health may also influence the efficiency of bacterial inoculations (Souza et al., 2015).

In the 2011/2012 season, at 30 or 90 DAP, no increases in dry weight was observed due to the inoculation of the diazotrophs compared to an untreated control without N-fertilizer. However, at 60 DAP, all the diazotrophs enhanced dry weight relative to the untreated control without N, the treated plants were equivalent to the untreated control with 33%N-fertilizer. Diazotrophic bacteria operate in the rhizosphere of host plants. The host root system has to be established first, before the bacteria can start fixing N, and otherwise stimulating plant growth (Sood, 2003). Therefore, we speculate that diazotrophic bacteria take some time to establish themselves in a host rhizosphere, which has to grow in the first place. A further dynamic is that root exudates change with changes in plant development (Micallef et al., 2009), which in turn will affect rhizosphere bacteria, including the diazotrophs. As a plant reaches end of life cycle, the root exudates diminish (Micallef et al., 2009), and we suggest that this will be linked with diminishing diazotrophs activity.

The positive effect of seed inoculation with diazotrophic bacteria on shoot dry weight and yield of maize has been reported by many researchers (de Salamone et al., 1996; Dobbelaere et al., 2003; Kennedy et al., 2004; Wu et al., 2005; Ferreira et al., 2013; Liu et al., 2015). Such an improvement may be attributed to the nitrogen-fixing and phosphate solubilizing capacity of these rhizobacteria, as well as their ability to produce growth promoting substances (Kloepper et al., 1989, 2004; Rodríguez and Fraga, 1999).

Three key results in the trials conducted here were that the diazotrophs enhanced seed germination, they took time to cause growth stimulation (>30 days), and that stimulation of the maize plants by the diazotrophs worked better if there was no N fertilizer application applied concurrently (**Table 3**).

The quantity of N fixed by biological nitrogen fixation (BNF) in cereal crops may be relatively small when compared to the application of inorganic fertilizer sources of N by commercial farmers. However, most resource-poor farmers cannot afford the cost of inorganic fertilizers, transport and application, estimated at more than 75% of small scale farmers in South Africa (Cedric and Nelson, 2014). For these farmers, BNF generated by diazotrophs could provide a substantial increase in N availability for their crops, at an affordable price, with a simple, accessible technology. The costs of application of a formulated diazotroph would be under US$5.00 per ha when applied as a seed treatment versus an estimated US$500 per ha for N applied as limestone ammonium nitrate. Therefore, it may be concluded that resource-poor farmers could benefit from the use of diazotrophic inoculants applied to maize crops in South Africa.

## Author Contributions

Both authors were involved in planning the experiments. Dr. MK conducted the research in the Lab, greenhouse and field data collection, analysis and final write up also done by Dr. MK. Editing of the manuscript was done by Prof. ML.

## Conflict of Interest Statement

The authors declare that the research was conducted in the absence of any commercial or financial relationships that could be construed as a potential conflict of interest. The reviewer MS and handling Editor declared their shared affiliation, and the handling Editor states that the process nevertheless met the standards of a fair and objective review.
